# 14-3-3 proteins—a moonlight protein complex with therapeutic potential in neurological disorder: in-depth review with Alzheimer’s disease

**DOI:** 10.3389/fmolb.2024.1286536

**Published:** 2024-02-05

**Authors:** Gholamareza Abdi, Mukul Jain, Nil Patil, Bindiya Upadhyay, Nigam Vyas, Manish Dwivedi, Radhey Shyam Kaushal

**Affiliations:** ^1^ Department of Biotechnology, Persian Gulf Research Institute, Persian Gulf University, Bushehr, Iran; ^2^ Cell and Developmental Biology Laboratory, Research and Development Cell, Parul University, Vadodara, Gujarat, India; ^3^ Department of Life Sciences, Parul Institute of Applied Sciences, Parul University, Vadodara, Gujarat, India; ^4^ Biophysics and Structural Biology Laboratory, Research and Development Cell, Parul University, Vadodara, Gujarat, India; ^5^ Amity Institute of Biotechnology, Amity University, Lucknow, Uttar Pradesh, India

**Keywords:** 14-3-3 protein, Alzheimer’s disease, tau protein, mutations, phosphorylation binding grove, protein-protein interaction

## Abstract

Alzheimer’s disease (AD) affects millions of people worldwide and is a gradually worsening neurodegenerative condition. The accumulation of abnormal proteins, such as tau and beta-amyloid, in the brain is a hallmark of AD pathology. 14-3-3 proteins have been implicated in AD pathology in several ways. One proposed mechanism is that 14-3-3 proteins interact with tau protein and modulate its phosphorylation, aggregation, and toxicity. Tau is a protein associated with microtubules, playing a role in maintaining the structural integrity of neuronal cytoskeleton. However, in the context of Alzheimer’s disease (AD), an abnormal increase in its phosphorylation occurs. This leads to the aggregation of tau into neurofibrillary tangles, which is a distinctive feature of this condition. Studies have shown that 14-3-3 proteins can bind to phosphorylated tau and regulate its function and stability. In addition, 14-3-3 proteins have been shown to interact with beta-amyloid (Aβ), the primary component of amyloid plaques in AD. 14-3-3 proteins can regulate the clearance of Aβ through the lysosomal degradation pathway by interacting with the lysosomal membrane protein LAMP2A. Dysfunction of lysosomal degradation pathway is thought to contribute to the accumulation of Aβ in the brain and the progression of AD. Furthermore, 14-3-3 proteins have been found to be downregulated in the brains of AD patients, suggesting that their dysregulation may contribute to AD pathology. For example, decreased levels of 14-3-3 proteins in cerebrospinal fluid have been suggested as a biomarker for AD. Overall, these findings suggest that 14-3-3 proteins may play an important role in AD pathology and may represent a potential therapeutic target for the disease. However, further research is needed to fully understand the mechanisms underlying the involvement of 14-3-3 proteins in AD and to explore their potential as a therapeutic target.

## 1 Introduction

The 14-3-3 protein family, is a group of homologous proteins that are widely expressed throughout the body. 14-3-3 term derived from homogenates of calf brain’s 14th fraction during starch gel electrophoresis (Pair and Yacoubian, 2021). These proteins exhibit high evolutionary conservation from higher-order mammals to plants (Zhu et al., 2016). Mammals harbor a collection of seven isoforms within the 14-3-3 protein family, namely, 14-3-3β, γ, ε, η, ζ, σ, and τ/θ. These isoforms represent both the phosphorylated and unphosphorylated variations of the 14-3-3 proteins (Ichimura et al., 1988). The phosphorylated versions of 14-3-3 and are represented by two isoforms. (Aitken et al., 1995). Each 14-3-3 isoform has nine alpha helices and can dimerize to form homodimers or heterodimers with the same or different isoforms. Upon dimerization of 14-3-3s, distinctive “W”-shaped amphipathic pockets become apparent, serving as the primary location for binding with partner molecules. Within these 14-3-3 dimers, the lower section of the “W” shape is created through an antiparallel interaction involving the first and second α-helices of one monomer, alongside the third and fourth α-helices of the second monomer (Sluchanko and Bustos, 2019).

Non-enzymatic 14-3-3 protein function hinges on PPIs with numerous binding partners, and while doubly phosphorylated proteins with mobility to access both amphipathic grooves in 14-3-3 dimers exhibit heightened affinity, 14-3-3s lack enzymatic activity (Yaffe et al., 1997; Pennington et al., 2018). These phosphorylation-dependent binding partner motifs are not necessary for all interactions with 14-3-3s, and some 14-3-3 interactions do not even rely on phosphorylation (Ottmann et al., 2007). Through protein-protein interactions (PPIs), 14-3-3 proteins function as molecular adaptors. Upon binding to a client protein, 14-3-3s facilitate a structural alteration in the binding companion, or they mediate the interaction between two distinct proteins. These conformational alterations affect the binding partner’s enzymatic activity, reveal, or conceal localization motifs that have an impact on the binding partner’s subcellular localization. The initial demonstration of 14-3-3 regulation involved hydroxylase of TYR and TRP group enzymes, leading to the genetic abbreviation “YWHA” (Ichimura et al., 1988). Participating in a variety of protein-protein interactions (PPIs), 14-3-3 proteins assume pivotal roles as central nodes in numerous signaling pathways. This involvement spans across a range of cellular activities, encompassing apoptosis, cellular trafficking, modulation of cytoskeletal dynamics, and neural plasticity. Consequently, the involvement of 14-3-3s spans a broad array of diseases, encompassing cancer, metabolic disorders, immunological issues, and neurological conditions (Fan et al., 2019).

The ability of 14-3-3 proteins to determine the intracellular positioning of protein substrates has led to various hypotheses concerning their presence in protein aggregates: 1) these proteins might have a safeguarding role by containing harmful pathogenic proteins, 2) they could become confined within aggregates, resulting in their own functional impairment, and 3) they might promote the creation of protein aggregates that subsequently induce neurotoxic effects. Beyond their role in protein sorting, it is recognized that 14-3-3 proteins also enhance the stability of their bound partners, safeguard phosphorylated forms of target proteins, and regulate the enzymatic activity of substrates (Shimada et al., 2013). Malfunction of these functions could likewise contribute to the onset of neurodegenerative disorders.

## 2 14-3-3 proteins: an overview

### 2.1 Structural features and classification

The structure of 14-3-3 protein complex, there are 9 antiparallel alpha helixes which are labeled from helix A to helix I, show an amphipathic manner and are connected by a flexible loop (Yang et al., 2006). In the structure, two identical subunits come together by hydrophobic interactions to form a stable functional complex. The helix G and helix G’ are responsible for the symmetrical dimer which generates a cup-like shape (Yang et al., 2006). Dimeric ubiquitous protein 14-3-3 contains approx. 233–246 conserved sequences of amino acids and each helix in a 14-3-3 protein typically contains approximately 20–25 amino acids. A study by Aitken et al., 1995, analyzed the crystal structure of human 14-3-3ζ, which revealed the lengths of individual helices. Following this study, helices αA, αB, αC, αD, αE, αF, αG, αH, and αI in 14-3-3ζ contain 22, 15, 24, 17, 23, 21, 23, 19, and 21 amino acids, respectively. These statistics are based on the primary sequence and the crystallographic data of the protein (Santo et al., 2020). On the basis of protein sequencing, molecular cloning, genetic analysis, functions, tissue distribution, and expression patterns each isoform has its unique profile. Some isoforms are ubiquitous and some are tissue-specific. Table 1 gives general details about each isomer including the positional difference in sequences, isoelectric point and molecular weight. Table 2 depicts the distribution of isoforms among different organisms.

**TABLE 1:** General details about each isomer with Molecular weight, Isoelectric point, and the positional difference in sequence.

S. No.	Name of isoform	Molecular weight (kDa)	Isoelectric point (PI)	The positional difference in sequence			
				49th	93rd	123rd	153rd
1	14-3-3 β	28.7	4.7	Alanine	Glutamine	Methionine	Glutamic acid
2	14-3-3 γ	27.2	5.1	Tyrosine	Leucine	Tyrosine	Alanine
3	14-3-3 ε	28	4.9	Tyrosine	Leucine	Lysine	Lysine
4	14-3-3 η	28	5.3	Tyrosine	Leucine	Lysine	Lysine
5	14-3-3 ζ	28	4.7	Lysine	Isoleucine	Glycine	Phenyl alanine
6	14-3-3 σ	28.7	4.9	Lysine	Glutamine	Methionine	Glutamic acid
7	14-3-3 τ	28.7	4.9	Lysine	Isoleucine	Glycine	Phenyl alanine

**TABLE 2:** 14-3-3 isoforms in various organisms.

No.	Organism name	Total number of isoforms	14-3-3 β	14-3-3 γ	14-3-3 ε	14-3-3 η	14-3-3 ζ	14-3-3 σ		14-3-3 τ
1	Mammals (mice)	7^a^	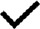	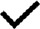	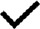	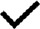	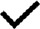	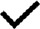	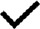	
2	Birds (chicken)	7^a^	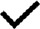	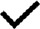	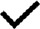	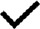	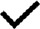	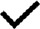	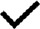	
3	Fishes (zebrafish)	7^a^	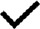	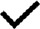	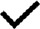	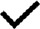	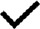	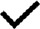	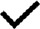	
4	Insects (*Drosophila melanogaster*)	2^a^	—	—		—		—	—	
5	Yeasts (*Saccharomyces cerevisiae*)	2^a^	In yeast two known isoforms of the 14-3-3							
			1) Bmh1 (known as YWHA1 or SGT1)							
			2) Bmh2 (known as YWHA2 or SGT2)							
6	Plants (*Arabidopsis thalian*a)	13^a^	Designated as 14-3-3a to 14-3-3n							

^a^ The number of isoforms may vary among different species

#### 2.1.1 Phosphorylation binding grove

In 14-3-3 proteins, a region called the phosphorylation binding grove (PBG) interacts with phosphorylated serine or threonine residues. They are clusters of residuals from the helical E, F, G, and H. The PBG is a group of amino acids that forms a pocket or groove and is found on the protein’s C-terminus. The negatively charged phosphate group of the phosphorylated residue interacts with the positively charged amino acids that line the pocket. Unphosphorylated residues are not able to attach to the PBG, which is selective for phosphorylated serine or threonine residues. The PBG is a highly conserved region of the 14-3-3 protein family. This suggests that the PBG is essential for the function of 14-3-3 proteins. Mutations in the PBG can disrupt the binding of 14-3-3 proteins to phosphorylated residues and can lead to a variety of cellular defects (Halskau et al., 2009).

#### 2.1.2 C terminal extensions

A group of amino acids called the C-terminal extensions are found at the end of 14-3-3 proteins. The fundamental role of 14-3-3 proteins, which is to bind to phosphorylated proteins, does not require these extensions. 14-3-3 proteins often have C-terminal extensions made up of 15–40 amino acids. Sequences of these extensions vary significantly across different 14-3-3 proteins as well as within the same protein. The binding affinity of 14-3-3 proteins for their ligands is hypothesized to be influenced by the sequence of the C-terminal extensions (Santo et al., 2020).

#### 2.1.3 N terminal helix

The short, amphipathic N-terminal helix of the 14-3-3 protein is crucial for membrane interaction and protein binding. The 12 amino acids that make up the helix are heavily concentrated in hydrophobic residues on one side and hydrophilic residues on the other. The helix can interact with hydrophobic as well as hydrophilic sites on other proteins to its asymmetry. This is also known as a helix -A and it also contributes to dimerization, and intracellular interactions and plays an important role in post-translation modifications by undergoing the process of site-specific phosphorylation and acetylation (Cho and Park, 2020).

### 2.2 Functions of 14-3-3 moonlight protein

**FIGURE 1:**
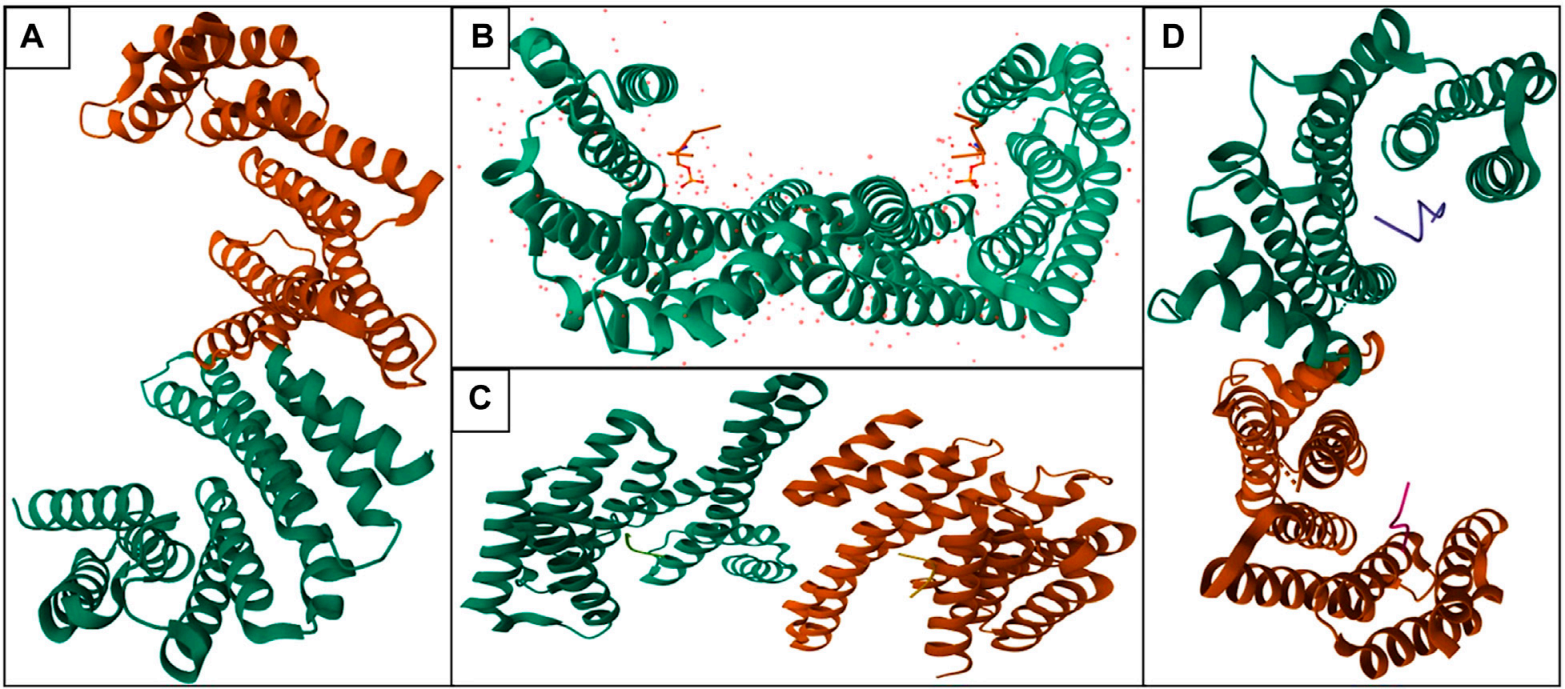
Structure of various isoforms—14-3-3 proteins in human (Orange color: Chain A and Green color: Chain B; Homodimer of 14-3-3 protein): **(A)** 14-3-3 protein beta (PDB ID: 2BQ0), **(B)** 14-3-3 protein epsilon (PDB ID: 2BR9), **(C)** 14-3-3 protein eta (PDB ID: 2C63), **(D)** 14-3-3 protein sigma (PDB ID: 4FL5).

The conserved moonlight protein 14-3-3 has not had a solo function but it could perform countless functions including cell cycle progression, programmed cell death, protein synthesis, cell signaling, or signal transduction, cell division, and many more (Santo et al., 2020). The phenomenon of 14-3-3 is not only observable in humans (Figure 1) but also in plants, yeast, *drosophila*, and *C. elegance*. 14-3-3 can regulate the other proteins by binding with them. 14-3-3 can enhance and inhibit the function of various proteins; in humans, 14-3-3 has various phosphate binding sites like RSx [pS/pT]xP and Rxxx [pS/pT]xP (Gardino and Yaffe, 2011; Stevers et al., 2017).

#### 2.2.1 Cell cycle progression

Another instance is mammalian cells can express three different types of proteins named Cdc25a, Cdc25b, and Cdc25c; these all are connected in G2/M progression. Cdc25b can activate CDK/cyclinB which phosphorylates cdc25c to create a binding site for the second mitotic kinase p1k1, which is turned to cdc25c by phosphorylation. Phosphorylation occurs on a single binding site especially in humans at 323rd residual of serine for cdc25B and at s216 of cdc25C. 14-3-3 can inhibit cdc25b and cdc25c.14-3-3 and bind to *Xenopus* cdc25c is responsible for the 2-fold reduction in its phosphatase activity. 14-3-3 can also block cdc25b and cdk1 binding by blocking cdk-1/cyclin B. 14-3-3 have also ability to stimulate the process of meiosis by enhancing cytokinesis activity. Specifically, 14-3-3 sigma is involved in the cytokinesis process (Gardino and Yaffe, 2011). A brief overview of functions with isoforms is given in Table 3.

**TABLE 3:** Isoforms—function and location of protein 14-3-3.

S. No.	Name	Location	Function	References
1	14-3-3 β	Cytoplasm - regulates signaling pathways and protein transport	Apoptosis, cell cycle regulation, and protein trafficking. It interrelates with several key proteins, like Raf-1 kinase and Bad, to modulate their activity and localization	Aghazadeh and Papadopoulos (2016)
				Fu et al. (2000)
				Hermeking and Benzinger (2006)
2	14-3-3 γ	Predominantly present in the cytoplasm and can translocate to the nucleus in response to specific cellular signals	Regulation of neuronal function, cell cycle control, and apoptosis, in CSF that can use as a marker for sporadic Creutzfeldt-Jakob disease. It interacts with diverse proteins, including Cdc25 phosphatases, Bax, and various kinases, to enhance their activity and subcellular localization	Cornell and Toyo-Oka (2017)
				Hermeking and Benzinger (2006)
3	14-3-3 ε	cytoplasm and the nucleus	Neuronal signaling, cell cycle regulation, apoptosis, and. It deals with proteins like c-Jun, Bim, and Bad to control their function and localization, responsible for RAS 1 signaling	Fu et al. (2000)
				Hermeking and Benzinger (2006)
				Matitau et al. (2013)
4	14-3-3 η	cytoplasm	less well-characterized compared to other isoforms. However, it has been suggested to be involved in neuronal development and neurodegenerative diseases	Cornell and Toyo-Oka (2017)
5	14-3-3 ζ	cytoplasm and the nucleus	Cell signaling, cell cycle regulation, and apoptosis, neurological disorders. It interacts with various proteins, such as Akt, PKC, and GSK3, to regulate their activity and subcellular localization, it can bind and regulate the activity of tyrosine hydroxylase	Aghazadeh and Papadopoulos (2016)
				Hermeking and Benzinger (2006)
6	14-3-3 σ	primarily located in the cytoplasm and has been reported to translocate to the nucleus in response to specific cellular signals	Cell cycle regulation (Essential for proper G2 checkpoint function) and tumor suppression (breast cancer). It interacts with proteins involved in cell cycle checkpoints, DNA damage response, and apoptosis, such as p53, cdc2, and Chk1	Hermeking and Benzinger (2006)
7	14-3-3 τ	The cytoplasm of neurons, but it can also translocate to the nucleus under certain conditions	Associated with neuronal function and is involved in regulating processes such as neurotransmitter release, synaptic plasticity, and neurodegeneration. It interacts with various neuronal proteins, including tyrosine hydroxylase and protein kinase C	Fu et al. (2000)

#### 2.2.2 Apoptosis

14-3-3 have the propensity to create integration with the BH-3 domain that contains proteins BAD, BAX. Exclusively BH-3 domain has a binding target of 14-3-3 on the IL-3-dependent survival mechanism in flj-12 in the hematopoietic cell line. This protein is vital for the activation of the BAD protein. 14-3-3 can also create phosphorylation in additional residues in BH-3 domain that led to permanent deactivation of it. Apart from this JNK directly phosphorylates 14-3-3 which leads to cell death (Gardino and Yaffe, 2011).

#### 2.2.3 Anti-apoptosis

In many cases, pro-apoptotic substances can be sequestered or inhibited by 14-3-3 proteins, which have anti-apoptotic effects. For instance, when the pro-apoptotic protein BAD protein is phosphorylated at certain locations by survival kinases, 14-3-3 proteins can bind to it and suppress its function, preventing it from interacting with members of the pro-survival Bcl-2 family. Anti-apoptotic proteins like Bcl-2 and Bcl-XL can bind with and stabilize 14-3-3 proteins, limiting their breakdown and fostering cell survival (Gardino and Yaffe, 2011).

## 3 Role of 14-3-3 proteins in neurological disorders

**FIGURE 2:**
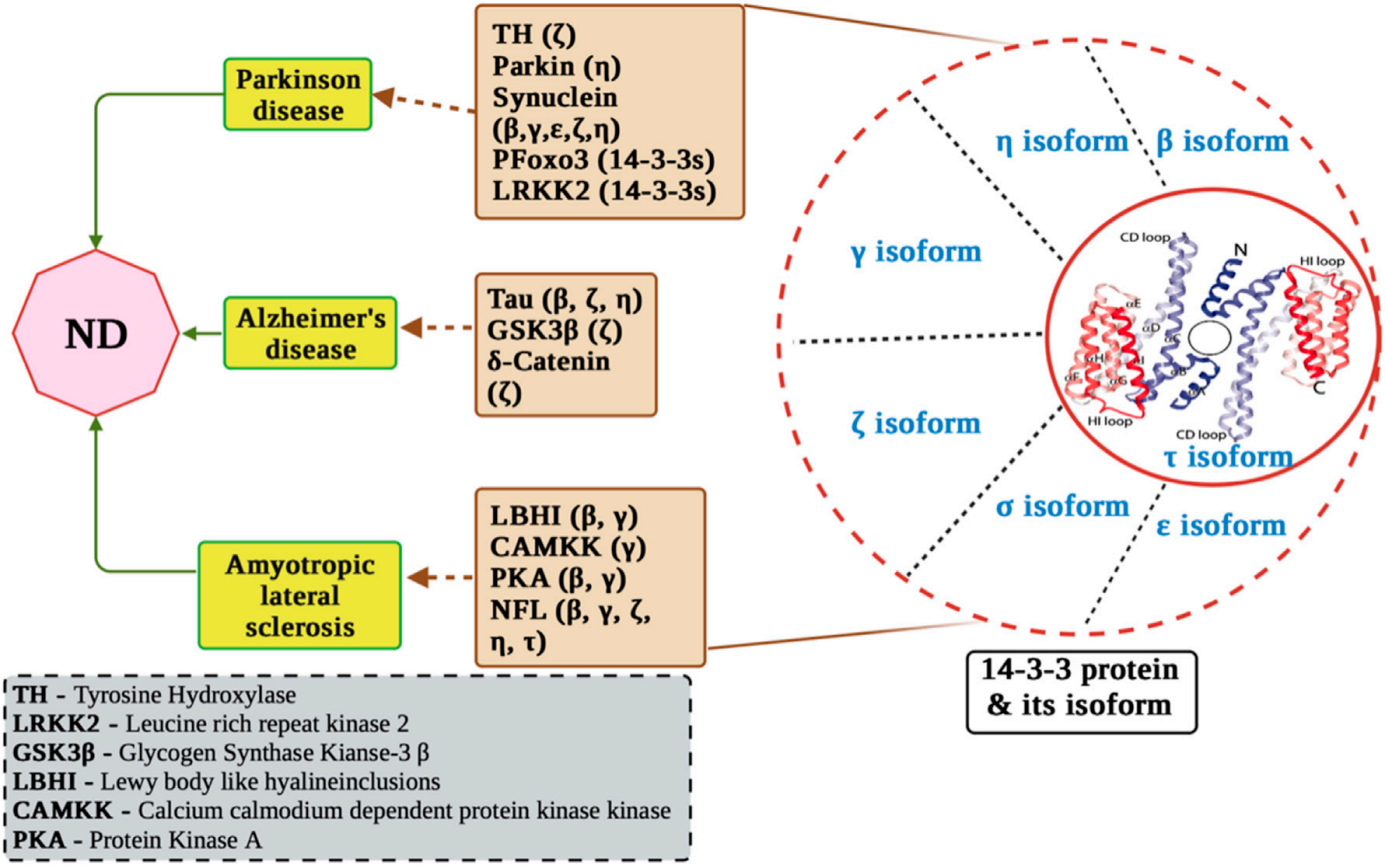
Molecular mechanisms of 14-3-3 proteins in neurological disorders. The figure displays the involvement of 14-3-3 proteins in Alzheimer’s disease pathogenesis, including their interactions with key proteins such as tau, amyloid-beta, and presenilin. In addition, it displays interaction of 14-3-3 with ALS related proteins and PD related proteins.

In both normal development and adulthood, 14-3-3 proteins assume varied physiological functions and engage with numerous substrate proteins, as supported by studies such as those by C. Mackintosh et al., in 2004 and Aitken, 1996 in 1996. Moreover, multiple lines of evidence highlight the significance of 14-3-3 proteins as crucial targets within neuropathological processes Figure 2 (Steinacker et al., 2011; Foote and Zhou, 2012).

14-3-3 proteins are detectable in cerebrospinal fluid in different neurodegenerative conditions like multiple sclerosis (Colucci et al., 2004), Creutzfeldt-Jakob disease (Hsich et al., 1996; Shiga et al., 2006), and HIV-associated neurodegeneration (Morales et al., 2012). Furthermore, 14-3-3 proteins function as biomarkers for neurological disorders characterized by substantial neuronal damage in the brain, such as acute stroke (K. Fujii et al., 2005) and subarachnoid hemorrhage (R. Siman et al., 2011). These observations suggest that their presence in cerebrospinal fluid may signify brain tissue destruction and the release of normal cellular proteins into the fluid (Siman et al., 2008; Goto et al., 2009). Moreover, 14-3-3 proteins are found within lesions and protein aggregates specific to each disease in the brain, and numerous studies have uncovered interactions between 14-3-3 and target proteins that regulate pathological processes (Steinacker et al., 2011; Foote and Zhou, 2012). This reinforces the concept that, beyond their role as overall indicators of tissue damage, 14-3-3 proteins actively participate in the development of ND. Given their potential influence on protein subcellular localization, the identification of 14-3-3 proteins within protein aggregates have sparked various hypotheses: 1) 14-3-3 proteins might offer protection by isolating toxic pathogenic proteins. 2) Sequestration of 14-3-3 proteins into aggregates could lead to their own functional impairment. 3) 14-3-3 proteins might aid in the formation of protein aggregates that subsequently induce neurotoxicity. Apart from their role in protein sorting, 14-3-3 proteins are acknowledged for stabilizing binding partners, preserving phosphorylated target protein forms, and controlling substrate enzyme activity; the breakdown of these functions might contribute to neurodegenerative diseases, as discussed below with specific examples.

### 3.1 Parkinson’s disease

PD, an age-related neurodegenerative condition, involves the loss of dopaminergic neurons in the substantia nigra pars compacta (M. R. Cookson et al., 2010). Clinical signs of PD include bradykinesia, tremor, postural instability, and progressive rigidity (Hughes et al., 1992). Lewy bodies, abnormal protein aggregations that form in neurons and are a pathological feature of Parkinson’s disease (PD), which was once thought to be rare, is now the fastest-growing neurological scenario in the world and has a high social and financial impact (Yang et al., 2020). In 1990, there were an estimated 2.5 million people with PD, and by 2015, that number had more than doubled to 6.2 million (Dorsey et al., 2018). By 2040, that number is projected to double once more to 12.9 million due to population ageing alone (Dorsey et al., 2018). Importantly 14-3-3s are found in LBs and are implicated in the pathophysiology of Parkinson’s disease (PD), interacting and co-localizing with various proteins like parkin124, α-Syn, and LRRK2 (Kawamoto et al., 2002). They are also found in other neurodegenerative disorders that include parkinsonian syndrome, like hereditary spastic paraplegia brought on by mutations in SPG11 (Faber et al., 2018). As PD incidence currently outpaces the rate of aging and is disproportionately on the rise in recently industrialized areas of the world, additional factors (such as pesticides, chemicals, air pollution, and decreased smoking) may cause that number to increase even further. The majority of 14-3-3 proteins can involve with α-synuclein, a protein that plays role in MAPK pathway regulation, thus holding significance in dopamine synthesis (Perez et al., 2002). Interactions between 14-3-3 (β and ε isoforms) and α-synuclein take place within both cytosolic and membrane fractions of rat brain homogenates (Ostrerova et al., 1999). Notably, co-immunoprecipitation of 14-3-3 and α-synuclein has been observed in the mammalian brain (Xu et al., 2002). *In vitro*, the 14-3-3η isoform significantly affects the aggregation products and kinetics of α-synuclein by binding to α-synuclein oligomers. Increased expression of the 14-3-3η isoform results in decreased α-synuclein toxicity in cellular models (Plotegher et al., 2014). A potential mechanism involves sequestration of 14-3-3 protein by associating with α-synuclein, causing 14-3-3 functionality loss, contributing to PD pathogenesis. Furthermore, the 14-3-3ζ isoform activates tyrosine hydroxylase (TH), a pivotal enzyme in the biosynthesis of catecholamine (Wang et al., 2009). The 14-3-3η binding to PARKN, a UBQE3 ligase, promotes protein degradation, and contributes to PD (Sato et al., 2006). Additionally, 14-3-3 proteins interact with LRRK2 and phosphorylated FOXO3a. FOXO3a localizes in Lewy bodies, and a proposed hypothesis suggests a cell survival-promoting complex involving FOXO3a, α-synuclein, and 14-3-3 proteins (Paisán-Ruíz et al., 2004; Kim et al., 2009). According to recent research, the binding of 14-3-3η is signalled by PKA-mediated phosphorylation of Ser955 in SPG11/spatacsin. It is still unclear if 14-3-3 dysregulation causes or results in LB formation. 14-3-3s co-localization in LBs, their altered phosphorylation in PD models and patients, and their acknowledged neuroprotective effects in several disease models leads to the speculation that 14-3-3s are likely to play a crucial role in the pathophysiology of PD, and their regulation may represent a possible strategy for future therapies (Terheyden et al., 2021). 14-3-3 proteins may influence the aggregation and toxicity of alpha-synuclein. These interactions could potentially affect the progression of PD by modulating protein misfolding and cellular responses. Dysregulation of 14-3-3 protein function might contribute to the disruption of essential cellular processes, impacting neuronal survival and function (Giusto et al., 2021).

### 3.2 Huntington’s disease

The relationship between Huntington proteins and 14-3-3 proteins has garnered significant attention in the field of neurobiology. HD is a devastating neurological disorder characterized by the aggregation of mutant HTT and progressive neurodegeneration (Rojas et al., 2022). Studies have revealed that mutant HTT interacts with various cellular proteins, including 14-3-3 proteins (Shimada et al., 2013). Evidence suggests that the interaction between mutant HTT and 14-3-3 proteins may influence disease pathology. In HD, the binding of mutant HTT to 14-3-3 proteins may contribute to altered cellular signaling pathways, impaired protein homeostasis, and dysregulated neuronal function (Podvin et al., 2022). Moreover, studies have implicated 14-3-3 proteins in the modulation of HTT aggregation and toxicity (Omi et al., 2008). Within Huntington’s disease (HD), the degenerative progression primarily affects medium spiny striatal neurons and has a relatively milder impact on cortical neurons. Notably susceptible are the GABAergic and enkephalin neurons situated in the basal ganglia, leading to early dysfunction and chorea development. Limited data on 14-3-3 protein levels in HD or its models, coupled with diminished 14-3-3 binding to GABA (B) R1 receptor subunits, implies its role in neurodegeneration-related pathophysiology (Cepeda et al., 2004). In HD, increased GABA receptor expression aligns with the hypothesis of diminished 14-3-3 interaction, which is essential for GABA receptor targeting from the ER to the cell membrane through binding to huntingtin-associated protein 1, disrupted in HD (Sarkar and Rubinsztein, 2008; Twelvetrees et al., 2010). The engagement of 14-3-3 proteins in overseeing the initiation of autophagy via energy-dependent mechanisms has been noted. Specifically, 14-3-3 proteins bind to phosphorylated raptor, a result of AMPK activation, leading to mTORC1 inactivation (Lee et al., 2010). Additionally, 14-3-3 proteins govern autophagy via Beclin-1 and hVps34 (Wang et al., 2010; Pozuelo-Rubio, 2011). Noteworthy is the association between 14-3-3 proteins and energy-regulated autophagy and apoptosis induction, evidenced by disrupted PGC-1α-dependent protein expression, including 14-3-3 proteins, in HD (Jin and Johnson, 2010). Compelling evidence from transfection studies shows that 14-3-3 proteins are crucial for mutant huntingtin aggregate formation. In N2A cells, 14-3-3ε, 14-3-3ζ, 14-3-3η, and 14-3-3θ interacted with polyQ-containing huntingtin’s N-terminal fragment (FURLONG et al., 2000). Suppression experiments using siRNA highlighted 14-3-3θ downregulation as the only effective approach to prevent mutant htt aggregation (Omi et al., 2008). Multiple connections exist between the transcriptional status of the huntingtin gene in Huntington’s disease (HD) and 14-3-3 proteins. Firstly, HDBP2 found in the promoter region of the IT15 gene coding for huntingtin, interacts with 14-3-3 proteins, possibly aiding HDBP2’s cytoplasmic-nuclear shuttling to regulate huntingtin expression (Tanaka et al., 2004; Sichtig et al., 2007). Secondly, the 14-3-3 protein control over histone deacetylases (HDACs) is implicated in HD (Gray, 2010). Investigating the interplay between Huntington and 14-3-3 proteins yield insights into HD’s molecular mechanisms and potential therapeutic avenues (complex interaction; targeting). According to research, 14-3-3 proteins may have an interaction with huntingtin, influencing both its toxicity and aggregation (Pair and Yacoubian, 2021). The abnormal accumulation of mutated huntingtin protein, which causes neuronal dysfunction and degeneration, is the hallmark of Huntington’s disease. The impact of 14-3-3 proteins on the dynamics of huntingtin may be involved in the development of HD (Schulte and Troy Littleton, 2011). Inaccuracies in the function of the 14-3-3 protein could upset the balance of the cell, affecting the survival ofneurons and intensifying the effects of the mutant huntingtin protein. Examining the intricacies of these interactions yields important information about the molecular processes behind HD (Steinacker et al., 2011).

### 3.3 Spinocerebellar Ataxia

Spinocerebellar ataxia (SCA) comprises a cluster of hereditary conditions marked by gradual cerebellar degeneration and related neural pathway deterioration, causing motor impairment and coordination issues. Autosomal dominant genetic mutations underlie SCA, resulting in a 50% likelihood of passing the mutated gene to each descendant (Ashizawa et al., 2018). Over 40 SCA subtypes exist, each linked to distinct genetic mutations, including prevalent types like SCA1, SCA2, SCA3 (Machado-Joseph disease), and SCA6 (van Prooije et al., 2021). The relationship between 14-3-3 proteins and Spinocerebellar Ataxia (SCA) pertains to their possible influence on the molecular processes that underlie this category of neurodegenerative illnesses. Progressive ataxia, which impairs balance and coordination, is a hallmark of SCAs. Recent studies indicate that 14-3-3 proteins might interact with particular proteins linked to different types of SCA (Umahara and Uchihara, 2010). 14-3-3 binds to ataxin-1, stabilises it, and encourages its build-up in transfected cells and transgenic flies (Chen et al., 2003). Notably, research has illuminated the interaction of 14-3-3 proteins with Spinocerebellar Ataxia Type 1 (SCA1). Ataxin-1 protein’s nuclear recruitment hinges on Akt-phosphorylation at Ser776 (Emamian et al., 2003), while 14-3-3 proteins stabilize phosphorylated ataxin-1, influencing both wild type and mutant forms (Chen et al., 2003). The interaction between ataxin-1/14-3-3 may either directly stabilise an ataxin-1 conformation that is resistant to degradation or it may obstruct access to other ataxin-1-interacting proteins that would otherwise aid in protein clearance (Fu et al., 2000). Observe that 14-3-3 interacts with both the unexpanded wild-type protein and the expanded mutant ataxin-1. Therefore, it is plausible that, in physiological locations 14-3-3 controls the clearance of ataxin-1. Since longer polyglutamine tracts improve ataxin-1’s interaction with 14-3-3, further stabilising the mutant protein, this regulation becomes problematic upon CAG repeat expansion (Tzivion and Joseph, 2002). These findings underscore the roles of both 14-3-3 proteins and Akt in influencing neurodegeneration. Human brain autopsy studies on SCA1 revealed the co-occurrence of expanded polyglutamine and 14-3-3 proteins within neuronal nuclei (Umahara et al., 2007), reinforcing 14-3-3’s involvement in SCA1-associated pathology and expanding our comprehension of molecular interactions in this disorder. The investigation into the interaction between ataxin-1 and 14-3-3 proteins in understanding Spinocerebellar Ataxia Type 1 (SCA1) has been explored. Research by Chen et al. indicated the isoforms of 14-3-3 proteins binding to ataxin-1 in certain conditions (Chen et al., 2003). This interaction is influenced by ataxin-1’s S776 phosphorylation site and is enhanced with longer polyglutamine stretches, stimulating the accumulation of ataxin-1 (82Q)-S776 and nuclear inclusion formation. The neurotoxicity of ataxin-1 is mediated by 14-3-3 proteins, which stabilize phosphorylated ataxin-1 (de Chiara et al., 2009). While PI3K/Akt signaling-mediated ataxin-1-S776 phosphorylation associates with SCA1 fly model degeneration, the specific kinase responsible and predominant 14-3-3 isoform involved remains uncertain (Jorgensen et al., 2009). The link between SCA and 14-3-3 proteins suggest their role in SCA pathogenesis; further research is essential to detail their mechanisms and potential as therapeutic targets.

### 3.4 Amyotrophic lateral sclerosis (ALS)

ALS is a neurodegenerative disorder that progressively impacts motor neurons in the brain and spinal cord, causing muscle weakening, paralysis, and loss of speech, swallowing, and breathing abilities, often marked by abnormal protein aggregate buildup (Keon et al., 2021).

A variety of clinical symptoms, including hyperreflexia, fasciculations, muscle atrophy, and involvement of the bulbar function. The molecular causes of ALS remain unclear despite the disease’s severe clinical manifestations, and mutations in the copper/zinc-dependent superoxide dismutase (SOD1) gene have only been found in a small fraction of familial ALS cases (Malaspina et al., 2000). The intricacy of neurodegenerative processes is highlighted by the possible involvement of 14-3-3 proteins in ALS, providing opportunities for additional investigation into the molecular mechanisms and possible therapeutic targets for this crippling illness (de Belleroche et al., 1996, Cruz-Sanchez et al., 1998; Cudkowicz et al., 1997. According to studies, 14-3-3 proteins may play a part in human neurodegenerative diseases that impact motor neurons, especially amyotrophic lateral sclerosis (ALS). The debilitating neurological condition known as ALS is typified by the selective degeneration of both upper and lower motor neurons. One group of proteins that have been implicated in ALS pathology is the 14-3-3 proteins. These molecules belong to a family of extensively preserved regulatory agents that hold pivotal functions in cellular signaling and operations. In the context of ALS, specific isoforms of 14-3-3 proteins have been found to interact with misfolded or aggregated proteins associated with the disease, such as superoxide dismutase 1 and TAR DNA-binding protein 43 (TDP-43) (Fan et al., 2019). Interaction with SOD1: Mutations in the SOD1 gene are known to be linked to a subset of familial ALS cases. In ALS patients with SOD1 mutations, the mutant SOD1 protein misfolds and forms toxic aggregates in motor neurons. Research has shown that certain 14-3-3 isoforms can interact with misfolded SOD1, potentially influencing its aggregation and toxicity (Okamoto et al., 2011). New studies have revealed that different 14-3-3 proteins—14-3-3b, c, f, h and e are present within ubiquitinated inclusions of anterior horn cells in sporadic ALS patients (Kawamoto et al., 2004). Elevated 14-3-3 mRNA levels are found in spinal cords of sporadic ALS individuals (Malaspina et al., 2000), with 14-3-3 proteins detected in ubiquitinated intraneuronal inclusions in ALS patients’ anterior horn cells (Kawamoto et al., 2004). 14-3-3 immunoreactivity is also seen in LBHIs of ALS patients with SOD1 gene deletion (Kawamoto et al., 2005) and their presence with SOD1 in LBHIs suggests 14-3-3’s role in their formation. 14-3-3 positivity in LBHIs of both sporadic and familial ALS underscores its contribution to ALS pathogenesis. Specific isoforms like 14-3-3f interact with misfolded proteins (Kaneko and Hachiya, 2006). While 14-3-3c and 14-3-3f might be oxidative damage targets in Alzheimer’s (Santpere & Ferrer, I. 2009), and neurofibrillary tangles display 14-3-3b and 14-3-3c labeling (Umahara et al., 2004). Furthermore, 14-3-3 proteins exhibit co-localization within Lewy bodies and glial cytoplasmic inclusions among patients with multiple system atrophy (Berg et al., 2003). Yet, the precise role of these interactions in disease progression remains uncertain. Notably, TDP-43, an aberrantly accumulated protein in ALS motor neurons, interacts with specific 14-3-3 isoforms, possibly influencing its localization and aggregation (Prasad et al., 2019). While these interactions in ALS pathology are captivating, their exact mechanisms require further elucidation (Mackintosh, 2004). Continued research into 14-3-3 proteins and other cellular elements in ALS pathology is crucial to enhance our comprehension and discover potential therapeutic targets in this intricate disorder.

### 3.5 Alzheimer’s disease

AD is a neurodegenerative, genetically intricate, age-related dementia marked by deteriorating memory. Damage to the brain’s neurons, which are nerve cells, is the root cause. Thinking, walking, talking, and all other human activities depend on the neurons in the brain (Griciuc and Tanzi, 2021). Neurons in regions of the brain involved in memory, language, and thought are the first to suffer damage in Alzheimer’s disease. As a result, issues with memory, language, and thought are frequently the first indications. Despite the fact that the affected person is experiencing new symptoms, the brain alterations that cause the condition start 20 years or more prior to the onset of symptoms (Knopman et al., 2021). According to the initial AD case report in 1906 (Perry, 2023), tau was considered a crucial MTAP (microtubule-associated protein), that stabilizes microtubules in neurons; however, AD is associated with tau hyper-phosphorylation, reducing its microtubule affinity (Ehrenberg, et al., 2017). NFT of AD patients have been found to contain 14-3-3 proteins, with 14-3-3 being the most immunoreactive (Umahara et al., 2004; McFerrin et al., 2017). By increasing GSK3’s affinity for tau, additional study has shown that 14-3-3 facilitates GSK3-dependent phosphorylation of tau (Yuan et al., 2004). Important to neuronal function 14-3-3s interact with Ab and tau and are found in Ab plaques and neurofibrillary tangles in human AD brains (Hashiguchi et al., 2000; Liao et al., 2004; Umahara et al., 2004; Sumioka et al., 2005). Genetic variants in the 14-3-3 genes are associated with AD risk. (Mateo et al., 2008; Mateo et al., 2008). 14-3-3s are important regulators of neuronal architecture: they stabilize synaptic spines and promote axonal and dendritic growth. Additionally, 14-3-3s regulate NMDA receptor (NMDAR) trafficking to the synapse and impact cerebellar and hippocampal long-term potentiation (Gohla and Gary, 2002; Gehler et al., 2004; Ramser et al., 2010; Yoon et al., 2012; Foote et al., 2015; Lavalley et al., 2016; Kaplan et al., 2017). Indeed, the functional 14-3-3 knock-out mice expressing the pan 14-3-3 inhibitor, difopein, show deficits mimicking several aspects of AD mouse models, including reduced spine density, reduced NMDAR subunits at the synapse, impaired long-term potentiation, and hippocampal-dependent learning and memory deficits 3u levels in aged rats correlate with decreased cognitive function. Recently, a dramatic 40% reduction in total soluble 14-3-3 protein levels in AD brains was observed, relative to age-matched controls, with a converse increase in total 14-3-3 levels in the insoluble fraction AD (Gannon et al., 2022). The brain protein δ-catenin, initially discovered in association with presenilin 1, is additionally connected to the 14-3-3ζ isoform (He et al., 2013). Apart from their unique interactions with proteins associated with neurodegenerative conditions, 14-3-3 proteins also exhibit a protective impact on dopaminergic neurons (Zhou et al., 1997). In fact, the 14-3-3 isoforms θ, γ, and decrease the cellular toxicity brought on by neurotoxins, which results in the death of dopaminergic cells (Betarbet et al., 2000; Yacoubian, et al., 2010). Furthermore, 14-3-3 proteins might play a role in aggresome formation, facilitating the chelation and breakdown of harmful oligomers and aggregates (Jia et al., 2014). Recently, 14-3-3 proteins were found to affect the autophagy process through their recognition of phosphorylated transcription factor EB (TFEB), a factor linked to neurodegenerative diseases (Xu, et al., 2019). Considering their involvement in age-related neurodegeneration, targeting 14-3-3 PPIs with therapeutic interventions could prove beneficial for disease management.

## 4 Molecular mechanisms of 14-3-3 proteins in Alzheimer’s disease

In Alzheimer’s, 14-3-3 proteins interact with key players like tau and amyloid beta, influencing their aggregation and misfolding, which are hallmarks of the disease. By deciphering the complex interplay between 14-3-3 proteins and Alzheimer’s-related proteins, researchers aim to unveil potential therapeutic targets for this neurodegenerative disorder.

### 4.1 14-3-3 proteins and amyloid beta pathology

**FIGURE 3:**
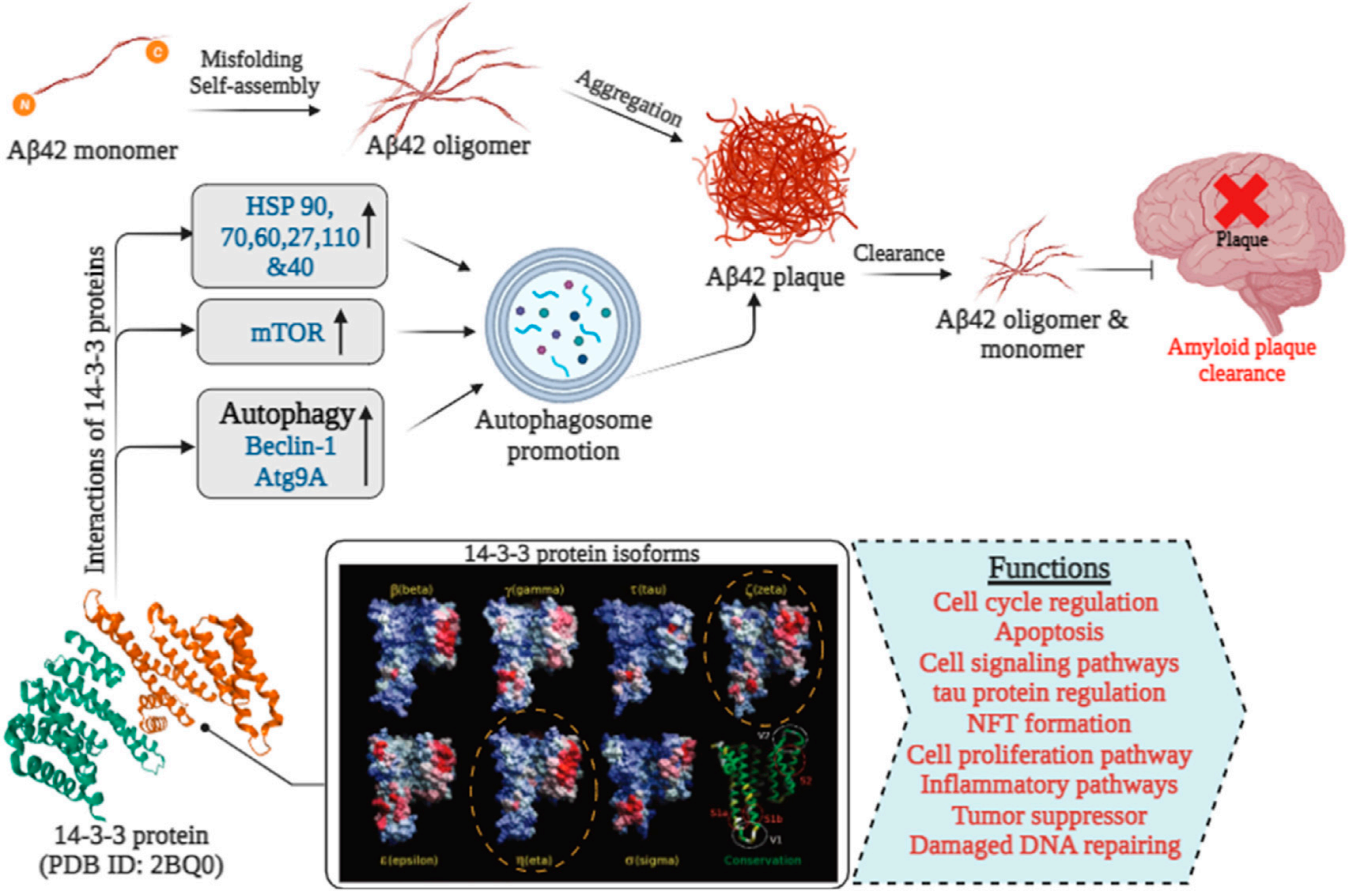
The role of 14-3-3 proteins in Aβ 42 clearance in Alzheimer’s disease [14-3-3 isoforms were extracted from (Yang et al., 2006)].

Aβ, a 4 kDa fragment derived from the APP, originates from a larger precursor primarily generated by neurons in the brain, as well as vascular and blood cells (including platelets), and to a lesser degree, astrocytes Figure 3. The increased hydrophobicity of the Aβ42 peptide compared to Aβ40 arises from the presence of an additional Ile-Ala dipeptide at its C-terminus (Yang and David, 2008). This specific structural alteration contributes to a greater affinity for interactions with the 14-3-3ζ protein. This primarily occurs in the β-strand-forming sections of Aβ40 and α-synuclein during their initial aggregation, disrupting β-sheet formation—the initial step in amyloid fibril creation. The effectiveness of 14-3-3ζ in suppressing fibril formation by Aβ peptides and A53T α-synuclein is influenced by multiple factors, similar to ten human small heat-shock proteins. Specifically, 14-3-3ζ interacts with L11-H21 and G29-V40 of Aβ40, and hydrophobic regions V3-K10 and V40-K60 of α-synuclein, occurring within amyloid fibril cores *in vitro* and isolated from disease-affected individuals.

Interactions between 14-3-3ζ and α-synuclein’s N-terminal regions align with interactions observed by in-cell NMR spectroscopy with other chaperones (Williams et al., 2021). Aβ arises from two sequential proteolytic cleavages of APP by β-secretase (BACE1) and γ-secretase (Blennow et al., 2006). In 1984, Aβ was first identified as a key component in meningovascular polymorphic deposits in Down syndrome patients. The majority of 14-3-3 target proteins possess binding motifs, including RSXpSXP (mode I) and RXY/FXpSXP (mode II), or -pS/pTX (1–2)–CO2 H (mode III), with X not being Pro. Notably, the core sequence of parenchymal Aβ plaque matches the perivascular component, with some differences. The APP gene sequencing confirmed Aβ as a product of APP enzymatic processing (Kang et al., 1987). Dense Aβ aggregates form neocortical neuritic plaques, a hallmark of aging and AD along with tau neurofibrillary tangles (NTFs) (Cras et al., 1991).

### 4.2 14-3-3 proteins and tau pathology

Tau and 14-3-3 proteins, both prominently found in the central nervous system, have attracted considerable interest because of their interplay. Hashiguchi et al. (2000), who employed a GST pull-down assay using glutathione-agarose beads coated with either GST or GST14-3-3ζ, provided the first indications of their direct interaction. The binding location was pinpointed to tau’s MTBD, with an estimated dissociation constant of 0.9 μM. Subsequently, sections containing pSer214 and pSer324 residues were identified as crucial for the interaction with 14-3-3 proteins (Joo et al., 2015; Sadik et al., 2009a; Sluchanko et al., 2009a). SPMR investigations demonstrated a tenfold higher binding affinity of phosphorylated tau to 14-3-3ζ, decreasing the Kd from 312 to 22 nM (Sadik et al., 2009a). Subsequent studies found that unphosphorylated 3R-tau binds roughly 3x more to 14-3-3ζ compared to unphosphorylated 4R-tau (Sadik et al., 2009b). However, the Kd evident in the phosphorylated 3R-tau/14-3-3ζ complex, as determined through gel electrophoresis, notably exceeds the value obtained from surface plasmon resonance by approximately 2 μM, despite consistent effects on binding affinity due to phosphorylation (Sluchanko et al., 2009b). Additionally, 14-3-3 binding modulates tau’s interaction with protein kinases, enhancing phosphorylation events and influencing neuronal processes (Yuan et al., 2004; Chun et al., 2004; Hashiguchi et al., 2000; Qureshi et al., 2013). Recently, NMR investigations examined the interaction between phosphorylated 4R-tau and 14-3-3σ, pinpointing pSer214 and pSer324 as primary sites. Titration experiments using PKA-phosphorylated (15N) Tau and 14-3-3 proteins indicated that the Kd value for the complex was 6.5+/-1.9 μM, with saturation occurring at a 1:2 ratio of Tau to 14-3-3 dimers (Joo et al., 2015). Microscale thermophoresis experiments indicated concurrent binding of pSer214 and pSer324 motifs in tau to a 14-3-3σ dimer, with monophosphorylated peptides showing larger Kd values than diphosphorylated ones (Joo et al., 2015; Sluchanko et al., 2009a; Sluchanko et al., 2009b), possibly influenced by tau concentration, phosphorylation state, and 14-3-3 dimer stability. The crystal structure of 14-3-3σ complexed with pSer214 and pSer324 peptides revealed an elongated structure with prevalent electrostatic interactions (Joo et al., 2015). Mutagenesis confirmed significance of Ser214, Ser324, and Ser356 in tau/14-3-3 interactions, affecting tau’s role in microtubule dynamics (Sluchanko et al., 2009a; Sluchanko et al., 2009b; Joo et al., 2015; Li and Paudel, 2016).

### 4.3 14-3-3 binding and cofilin accessibility

Cofilin serves as a critical regulator in both cytoskeletal and neuronal functions, maintaining the dynamic structure of the actin cytoskeleton crucial for processes like cell migration and synaptic function (Bamburg et al., 2021). Hyper-phosphorylation of cofilin contributes to synaptic dysfunction and induces oxidative stress via the PrPc-NOX pathway (Dirnagl et al., 1999). Kim et al. (2009) propose that oxidation of 14-3-3 under Alzheimer’s disease conditions releases SSH1, activating cofilin through dephosphorylation and promoting mitochondrial dysfunction. Additionally, studies by Rahman et al. (2014) reveal cofilin aggregation with Aβ oligomers in AD brain tissues, linking interactions with synaptic proteins and RanBP9-mediated cofilin actin rod accumulation, leading to mitochondrial dysfunction. Woo et al. (2015) demonstrate that reduced cofilin expression, achieved by downregulating RanBP9, protects against memory and learning defects in a contextual fear conditioning mouse model, underscoring the importance of cofilin activity levels in hippocampal learning and memory. In Tau-P301S mice, oxidation of 14-3-3 releases SSH1, activating cofilin and promoting tauopathies by competitively inhibiting tau-microtubule interactions (Yoshiyama et al., 2007). 14-3-3ζ regulates actin dynamics by interacting with phosphorylated cofilin, inhibiting its binding to F-actin. Cofilin and its kinase LIMK1 also bind to 14-3-3ζ, suggesting C-terminal interactions that impede cofilin-F-actin binding (Mizuno, 2013). Recent findings indicate 14-3-3ε and 14-3-3ζ binding to δ-catenin, activating the Rho GTPase-LIMK1 pathway. Loss of 14-3-3 proteins stabilizes δ-catenin, reducing LIMK1 activity, lowering p-cofilin levels, and promoting F-actin formation and spinogenesis in the actin regulatory pathways (Toyo-Oka et al., 2014). Therefore, the 14-3-3 proteins and their interaction with cofilin represent one of the many molecular pathways under investigation for potential therapeutic interventions in AD.

## 5 Therapeutic potential of 14-3-3 proteins in neurological disorders

Neurological disorders, encompassing a broad spectrum of conditions affecting the brain and nervous system, represent a significant global health burden. Despite advancements in understanding the molecular mechanisms underlying these disorders, effective therapeutic interventions remain limited. However, recent studies have illuminated the therapeutic possibilities associated with 14-3-3 proteins, a versatile group of regulatory proteins that are widely conserved, for tackling a range of neurological disorders. By comprehending the complex interactions of 14-3-3 proteins in neurological disorders, we seek to lay the groundwork for forthcoming therapeutic approaches that harness the versatile potential of these proteins, aiming to address and combat devastating neurological conditions, such as Alzheimer’s disease (AD).

### 5.1 Targeting 14-3-3 proteins as therapeutic strategy for AD with experimental and preclinical studies

As the above-mentioned topic suggests that, 14-3-3 protein, play crucial role in different neurological disorders and from this, here we discussed about the use of 14-3-3 protein as target for a therapeutic strategy in Alzheimer’s disease. In the context of AD, 14-3-3 proteins have been found to interact with several key players involved in AD pathology, including tau protein and amyloid-beta (Aβ) peptide (Sultana et al., 2009). For targeting 14-3-3 as a therapeutic, several approaches have been proposed such as, modulation of 14-3-3 interaction, enhancing 14-3-3 chaperone activity, gene therapy and targeted delivery.

Modulation of 14-3-3 interactions potentially modulate the activity of interaction of key proteins like tau, p53, HSP20 and LRKK2 with 14-3-3. The 14-3-3 dimer’s top rim, which is less conserved than the core channel and is where the pockets are found, is where 14-3-3 binds to AANAT (Aralkylamine N-acetyltransferase) and FT (Farnesyltransferase). AANAT initially recognized for its role in melatonin synthesis, has garnered attention due to its potential involvement in regulating amyloid beta levels and neuroinflammation. AANAT is also going to interact internally with FT, which associated with protein prenylation that add another complexity layer in complexity of tau tangles (Stevers et al., 2017). Another way, it is indicated that stabilization of 14-3-3’s interactive proteins are often preferable than inhibition as a strategy. Numerous instances of small compounds that form connections with both 14- 3-3 and interactive partners have been report as examples of this. They function as molecular glue in this way, improving selectivity chances. Through a combination of inhibition and stabilization strategies, various inhibition approaches have been explored. Bartel et al. (2014) demonstrated that the R18 peptide’s binding to 14-3-3 involves salt bridges between Glu and Arg residues, providing effective competition for both phosphorylation-dependent and -independent interactions. Similarly, targeting both types of interactions with R18 could potentially affect processes like tau aggregation, synaptic dysfunction, and neuroinflammation. (Bartel et al., 2014). Group of Ottmann and Grossmann in 2014, have been developed a strategy of macrocylcization of peptide that inhibits the 14-3-3ζ and *vir* factor of *Pseudomonas aeruginosa* Exoenzyme S (ExoS). By replacing Leu422 and Ala425 with S-configured non-natural amino acids cross-linked to a 12-methylene chain, they successfully developed a peptide with approximately a 30-fold increase in affinity. This modified peptide is designed to inhibit the T3S system, which is influenced by the ExoS factor, thereby potentially reducing neuroinflammation and slowing down the progression of Alzheimer’s disease (Glas et al., 2014). Regarding stabilizing 14-3-3 PPIs, a diterpene glycoside (Fusicoccane) from Phomopsis amygdali fungus was identified. In 1994, researchers uncovered a dual partnership between the regulatory segment of plasma membrane H^+^-ATPase (PMA) and 14-3-3 adapter proteins, forming a “molecular glue” that enhances interaction stability. Recent research on Fusicoccane unveiled its role in boosting platelet adhesion to von Willebrand factor by strengthening the interaction between 14-3-3 and the C-terminus of human glycoprotein (GP) Ibα (Santo et al., 2020). Another study from (Rose R. et al., 2010) indicates that, Epibestatin, Pyrrolidone, and Pyrazole have surface immobilized pocket with PMA2-CT52 and 14-3-3/PMA2 protein-protein interface. These immobilized pockets modify scaffold of pyrrolidone into a more rigid pyrazole ring, inhibit the protein protein interaction of tau tangles, and suppress the progression of AD (Rose R. et al., 2010).

14-3-3 proteins can modulate the activity of binding proteins by either inhibiting or promoting protein-protein interactions with other proteins (Pennington et al., 2018). In this manner, it interact with the HSP (heat shock protein) like chaperon and regulate their activity (Sluchanko and Gusev, 2017). Yano et al., ’s 2006 study showed that 14-3-3 not only prevented the aggregation of citrate synthase caused by heat but also collaborated with Hsp40/Hsp70 to reverse heat-aggregated citrate synthase and restore its solubility. The study highlighted that the γ-isoform of 14-3-3 notably delayed the heat-induced aggregation of rhodanase. This chaperone-like role of 14-3-3 paralleled that of HspB6 (Hsp20) (Yano et al., 2006). Through a thorough examination of 14-3-3ζ′s chaperone-like activity using various model protein substrates, it was observe that both monomeric and dimeric forms of 14-3-3ζ were incapable of impeding the aggregation of lysozyme induced by reduction. Surprisingly, rather than preventing aggregation, 14-3-3ζ actually facilitated the aggregation of this particular model substrate (Park et al., 2002). In inhibiting reduction-induced insulin aggregation and heat- and EDTA-induced alcohol dehydrogenase aggregation, chaperone-like function of 14-3-3ζ was remarkably effective, showing a concentration-dependent effect. Notably, the monomeric state of 14-3-3ζ displayed greater chaperone-like activity than its dimeric form in these situations (Sluchanko and Gusev, 2017). Phosphate ions do not inhibit the chaperone-like activity of 14-3-3, as evidenced by its ability to prevent aggregation of model proteins in 50 mM phosphate buffers (Sluchanko et al., 2014). In summary, the partial dissociation of 14-3-3 dimers reveals the disordered N-terminal region, increasing hydrophobicity and creating a favorable context for interacting with unfolded, misfolded, or immature client proteins. This mechanism substantially amplifies 14-3-3’s chaperone-like function, bolstering its role as a molecular chaperone.

In the realm of Alzheimer’s disease treatment, gene therapy emerges as a hopeful strategy, with the potential for the 14-3-3 protein to assume a pivotal function. By employing targeted delivery methods, gene therapy can introduce therapeutic genes into specific brain regions, while the 14-3-3 protein can act as a chaperone to facilitate the proper folding and functioning of target proteins, potentially alleviating Alzheimer’s pathogenesis. The study of Gu et al., 2020 indicates that, the downregulation of 14-3-3γ and 14-3-3η isoforms, in the frontal cortex of AD patients could potentially impair the anti-apoptotic function, leading to increased neuronal cell death and contributing to AD pathology. Therefore, the upregulation or activation of these isoforms was mainly conducted through various approaches such as gene overexpression, regulation of TF, modulation of mi-RNA or through designing therapy vectors with promoters or enhancers that enhance the process of transcription of 14-3-3γ and 14-3-3η (Gu et al., 2020).

Regarding preclinical investigations, a recent study by Yuanyuan Lu in 2022 examined 113 controls, 372 MCI patients, and 225 AD dementia patients using data from the ADNID. The research highlighted those increased concentrations of 14-3-3ζ in cerebrospinal fluid (CSF) were indicative of more pronounced cognitive deterioration, greater brain atrophy, reduced glucose metabolism, and the accumulation of Aβ over time in all subjects. These observations were made using genotyping, neuroimaging, and mass spectrometry assessments. This study’s findings underscore the potential of targeting 14-3-3ζ with biologics as a promising avenue for Alzheimer’s disease treatment (Lu, 2022). A study conducted by N. Huang in Brazil involved 46 patients with rapidly progressive dementia, categorized into definitive or probable Creutzfeldt-Jakob disease (CJD), possible CJD, and other diagnoses. Employing immunoblotting, the researchers identified the presence of 14-3-3 protein in cerebrospinal fluid (CSF), finding a strong association between its presence and CJD. Among cases classified as definitive or probable Creutzfeldt-Jakob disease (CJD), 82% tested positive for 14-3-3 protein in CSF, with only three patients (1 probable sporadic and 2 familial cases) yielding negative results. In the possible CJD group, 42% had positive 14-3-3 protein results in CSF. Notably, three individuals (13%) from the group with other diagnoses exhibited false-positive 14-3-3 protein results (Huang et al., 2003). To conclude, this investigation emphasizes the value of identifying 14-3-3 proteins in CSF as a diagnostic approach for CJD. Nonetheless, distinguishing CJD from other rapid progressive dementia sources in clinical settings is limited due to potential false positives in cases like AD. A limited number of biomarkers have undergone extensive study in Alzheimer’s disease (AD) and Lewy body dementia (LBD) to date. Despite conflicting results in prior studies on α-syn and pTau in cerebrospinal fluid (CSF), recent advances in seed amplification assays now permit the detection of α-syn brain proteinopathies in both CSF and skin (Koníčková et al., 2022). These assays demonstrate high accuracy in discriminating dementia with Lewy bodies (DLB) from control or AD patients (Bousiges et al., 2020). Similarly, previous research suggests that the core CSF biomarkers typically used to support AD diagnosis amyloid β peptide (Aβ1-42), total tau (tTau), and phosphorylated tau (pTau) offer limited diagnostic accuracy in distinguishing DLB from AD, as they are abnormal in approximately 25%–40% of DLB patients due to comorbid AD pathology (Lehmann et al., 2020). CSF proteome profiling aids in identifying changes across a wide spectrum of biological processes *in vivo* (Higginbotham L. et al., 2020). As seen in the AD field, such analyses contribute to defining the molecular mechanisms involved in disease pathogenesis and revealing promising biomarker candidates. According to the study by (Wesenhagen et al., 2020), CSF proteome profiling enables the identification of changes spanning various biological processes *in vivo*. This analysis, consistent with observations in the AD field, enhances our understanding of the molecular mechanisms in disease pathogenesis and identifies promising biomarker candidates. Therefore, additional preclinical studies and clinical trials are necessary to confirm the meaningful role of 14-3-3 proteins in the context of AD.

### 5.2 Challenges and future perspectives

The study of 14-3-3 protein in the context to AD has provided valuable insight into pathophysiological mechanisms which underlying AD. However, there are some challenges like the detection of 14-3-3 within CSF has demonstrated notable sensitivity and specificity in identifying Creutzfeldt-Jakob disease (CJD), its diagnostic accuracy for AD may not be as precise (Abu Rumeileh et al., 2017). As seen in the study mentioned, some cases of AD can yield false-positive results for 14-3-3 protein, which highlights the need for more specific and accurate biomarkers for the diagnosis of AD. One of the main key challenges, to understand how 14-3-3 protein levels change during the course of AD and how they correlate with disease progression and prognosis is essential (Candelise et al., 2020). Current immunoassay methods for 14-3-3 protein detection often struggle with identifying lower concentrations. The 14-3-3 protein capture assay, which exploits the strong binding affinity between the 14-3-3 protein and a chemically synthesized peptide with a phosphorylated recognition motif (Gogl et al., 2021), has been primarily used to assess 14-3-3γ levels in patients with Creutzfeldt-Jakob disease (CJD), covering both sporadic and non-sporadic cases. It has also been applied in patients with conditions such as Alzheimer’s disease (AD), dementia, Lewy body dementia (LBD), and Parkinson’s disease (PD) (Llorens et al., 2020). In this assay, the recognition sequence, connected to two alanine spacers and a cysteine residue at the N-terminus, is covalently linked to a maleimide-activated microplate. The resulting complexes of the peptide and 14-3-3 protein are quantified using an anti-14-3-3γ isoform antibody, followed by a peroxidase-conjugated secondary antibody and substrate (Yan et al., 2012). Additionally, immunoblot assays were employed to detect 14-3-3 protein levels, with semi-quantitative scoring based on a specific algorithm: no detectable signal, faint signal (+), moderate signal (++), strong signal (+++). Detection of the bound polyclonal antibody to the β isoform of the 14-3-3 protein (Santa Cruz Biotech, Santa Cruz, CA) was achieved using enhanced chemiluminescence (ECL) (Schmitz et al., 2016). While techniques are becoming more sensitive, for accurate diagnosis, the measurement of 14-3-3 should be considered alongside other biomarkers. Longitudinal studies were needed to explore the potential of 14-3-3 protein as a prognostic marker and to determine whether its levels can be used to monitor disease severity and response to treatments. Moreover, further investigation is needed to elucidate the exact role of 14-3-3 proteins in the development of AD. Research should delve into the interactions between 14-3-3 proteins and other molecules implicated in neurodegeneration, such as β-amyloid and tau proteins, potentially offering novel insights into therapeutic targets (Caterino et al., 2017). In genomics and proteomics, the therapeutic potential of targeting 14-3-3 proteins in AD remains uncharted. Exploring their expression, modulation, and activity could unveil novel therapeutic avenues for AD treatment.

## 6 Conclusion

The in-depth review on 14-3-3 proteins in the context of neurological disorders, with a focus on Alzheimer’s disease, has revealed several key findings. 14-3-3 proteins, once considered solely as regulatory proteins, have emerged as multifaceted moonlighting proteins with diverse roles in neuronal function, signal transduction, and protein-protein interactions. These proteins are prominently synthesized by the brain and hold a crucial role in various cellular functions, including cell survival, apoptosis, and synaptic plasticity. In AD, changes in 14-3-3 protein levels have been link to disease advancement and cognitive deterioration, suggesting their potential as biomarkers for early detection and prognostication. Investigating the specific molecular mechanisms by which 14-3-3 proteins modulate tau and amyloid-beta aggregation, neuroinflammation, and synaptic dysfunction could unlock potential therapeutic avenues. Longitudinal studies are essential to establish the temporal changes in 14-3-3 protein levels during disease progression and to explore their potential as predictive biomarkers for identifying individuals at risk of developing Alzheimer’s disease. The review presents exciting opportunities for the therapeutic targeting of 14-3-3 proteins in Alzheimer’s disease. Understanding their involvement in disease processes holds promise for advancing early diagnosis, identifying novel therapeutic targets, and developing potential therapeutic interventions. Continued research in this area will undoubtedly deepen our understanding of 14-3-3 proteins’ therapeutic potential, bringing us closer to effective treatments for Alzheimer’s disease and other neurological disorders.
